# Long Noncoding Ribonucleic Acid MSTRG.59589 Promotes Porcine Skeletal Muscle Satellite Cells Differentiation by Enhancing the Function of *PALLD*

**DOI:** 10.3389/fgene.2019.01220

**Published:** 2019-11-26

**Authors:** Long Li, Xiaofang Cheng, Ling Chen, Jingxuan Li, Wenzhe Luo, Changchun Li

**Affiliations:** ^1^Key Laboratory of Agricultural Animal Genetics, Breeding, and Reproduction of the Ministry of Education and Key Laboratory of Swine Genetics and Breeding of the Ministry of Agriculture, Huazhong Agricultural University, Wuhan, China; ^2^The Cooperative Innovation Center for Sustainable Pig Production, Huazhong Agricultural University, Wuhan, China

**Keywords:** porcine, skeletal muscle, satellite cell, long noncoding ribonucleic acid MSTRG.59589, *PALLD*

## Abstract

Skeletal muscle satellite cells are a class of undifferentiated mononuclear myogenic stem cells distributed between the myofibroblast and membrane basement. Since their development determines the development of skeletal muscles, knowledge of their proliferation, differentiation, and fate is vital for understanding skeletal muscle development. Increasing evidence have shown that long noncoding RNA (lncRNA) plays an important role in regulating the development process of satellite cells. Based on the results of our previous studies, we screened lncRNA MSTRG.59589, which is highly expressed in skeletal muscle tissue. In the present study, knockdown of MSTRG.59589 significantly inhibited satellite cell differentiation at various time points, whereas overexpression of MSTRG.59589 demonstrated opposite effects. An MSTRG.59589 knockdown cell model was constructed for transcriptome sequencing, and RNA sequencing analysis screened out a large number of differentially expressed genes. Gene Ontology and Kyoto Encyclopedia of Genes and Genomes enrichment analyses of these differentially expressed genes revealed that they are mainly enriched in actin cytoskeleton, muscle contraction, and other pathways related to muscle development. Mechanistic analyses showed that MSTRG.59589 could promote the differentiation process of skeletal muscle satellite cells by positively regulating the expression level of the target gene *PALLD*. This experiment lays a theoretical foundation for deeper studies on the mechanism of MSTRG.59589 in the differentiation of porcine skeletal muscle satellite cells.

## Introduction

The existence and development of skeletal muscles is a highly coordinated multistep biological process driven by paired box families, myogenic regulatory factors, and myocyte enhancer factor 2 (MEF2) family proteins (Berkes and Tapscott, 2005; Buckingham, 2006; Braun and Gautel, 2011; Yin et al., 2013). Pig skeletal muscle development is determined by the proliferation and differentiation of skeletal muscle satellite cells, which are a class of undifferentiated mononuclear spindle-shaped myogenic stem cells attached to muscle fibers and located between the basement membrane and muscle fiber sarcolemma (Mauro, 1961). After appropriate stimulation, such as physical activity, satellite cells are activated and begin to proliferate. Some satellite cells can continue to proliferate, differentiate, and fuse with existing muscle fibers (contributing the nucleus to muscle fibers), whereas some return to a static state and replenish the satellite cell pool through self-renewal (Wang and Rudnicki, 2011; Cornelison, 2018). The myofibroblast nucleus is mitotic and does not promote growth and repair (Ciciliot and Schiaffino, 2010). Considering the post-mitotic nature of myocyte nuclei, satellite cells are the only neonatal nucleus source of muscle fibers, making them essential for skeletal muscle growth and regeneration (Verdijk, 2014). Over the last few years, various signaling pathways controlling satellite cell differentiation have been identified. For example, activation of the Notch signaling pathway could inhibit muscle satellite cell differentiation (Mourikis et al., 2012; Kivela et al., 2016). The Wnt signaling pathway is indispensable in promoting the activation and differentiation of skeletal muscle satellite cells (Fujimaki et al., 2014). The mTOR signaling pathway regulates the differentiation of skeletal muscle satellite cells by controlling the expression of myogenic genes (Zhang et al., 2015). Activation of p38 signaling is correlated with satellite cell differentiation, while inhibition of p38 reversibly prevents differentiation (Charville et al., 2015). The regulation of satellite cell differentiation by several important signaling pathways, such as Notch, Wnt, and p38, has been revealed, and the related knowledge provides a basic framework for understanding the molecular mechanisms of satellite cell differentiation, which has remained largely unknown. Research on complex genetic networks, particularly that related to the effects of long noncoding RNAs (lncRNAs) on satellite cell differentiation, has just begun.

LncRNAs are a class of transcripts that are over 200 nucleotides long and structurally similar to mRNA but have low coding potential, expression level, and conservation (Diederichs, 2014). Many researchers have discovered that lncRNAs can participate in various biological processes. Several lncRNAs, such as lincMD1 (Cesana et al., 2011), H19 (Kallen et al., 2013), Malat1 (Watts et al., 2013), linc-YY1 (Zhou et al., 2015), lnc-mg (Zhu et al., 2017), and lncMAR1 (Zhang et al., 2018) have been identified in various species and found to play important roles in regulating skeletal muscle development. Accumulating evidence reveals that lncRNA regulates the proliferation and differentiation of satellite cells through a variety of mechanisms. For example, lnc133b attenuates the inhibitory effect of miR-133b on the target gene IGF1R by binding to miR-133b, promotes satellite cell proliferation, and inhibits satellite cell differentiation (Jin et al., 2017). The LncRNAs Snhg8 and Gm26917 are direct targets of the transcription factor FoxM1; the former can promote satellite cell proliferation by regulating the transcription of ribosomal protein genes, whereas the latter plays an important role in satellite cell survival (Chen et al., 2018). LINC00961 encodes a polypeptide called SPAR that regulates muscle regeneration by acting on lysosomes following starvation and amino-acid-mediated stimulation to inhibit mTORC1 activity (Matsumoto et al., 2017; Tajbakhsh, 2017). LncRNA SYISL may bind to the EZH2 protein in the PRC2 complex, leading to H3K27 trimethylation (H3K27me3) in the promoter region and a decline in the expression of the proliferation inhibitor P21; decreased expression of muscle-differentiation-related genes, MYOG, MYH4, and MCK, which promotes muscle cell proliferation and inhibits differentiation, may also be observed (Jin et al., 2018).

If an organism is treated as a machine and the skeletal muscle is a component of this machine, the satellite cell is equivalent to the production base of the component, and lncRNA is the worker in the production process of the component. When the component is old or must be replaced with a larger part, the production base will produce parts; in this process, the participation of workers is indispensable. Therefore, skeletal muscle satellite cells and lncRNAs are essential for muscle growth and development. However, although thousands of lncRNAs have been identified by techniques such as RNA sequencing (RNA-seq), only a few lncRNAs have been identified to play roles in the proliferation and differentiation of skeletal muscle satellite cells. The detailed molecular mechanisms of lncRNAs in regulating skeletal muscle satellite cell differentiation remain unclear (Zhao et al., 2015; Zou et al., 2017a; Zou et al., 2017b; Kern et al., 2018). At present, most studies on lncRNAs participating in the development of satellite cells have focused on humans, mice, and other model animals (Cabili et al., 2011). Given that lncRNAs are poorly conserved and specifically expressed in tissues and species, their biological functions in skeletal muscle and their satellite cells from other organisms cannot directly prove that they also have the same functions in pigs. Therefore, in the present study, we characterized the lncRNA MSTRG.59589, which is highly expressed in porcine skeletal muscle, and carried out its knockdown and overexpression, in differentiated porcine skeletal muscle satellite cells to identify the expression changes of differentiation markers and the phenotype transformation of satellite cells. We then utilized RNA-seq data to analyze the potential target genes and pathways probable molecular mechanisms of the lncRNA involved in the differentiation of satellite cells. We aim to provide a theoretical basis for analyzing the role of lncRNA in porcine skeletal muscle satellite cell differentiation and its effects on the growth and development of porcine skeletal muscles.

## Materials and Methods

### Isolation, Culture, and Differentiation of Porcine Skeletal Muscle Satellite Cells *In Vitro*

Porcine skeletal satellite cells were isolated from large white male pigs less than 7 days old. To put this experiment simply, the muscle of neonatal pig hind limb were collected and washed with PBS supplement with 1% antibiotic-antimycotic (AA) (Gibco, USA, Cat#15240-096), then connective tissue and adipose tissue were removed and the sterile muscle tissues were minced into small pieces, the tissues were dissected and digested with collagenase II (Gibco, USA, Cat#17101-015) at 37°C for 2.5 h in water bath shaker, Dulbecco's modified Eagle medium (DMEM) (Gibco, USA, Cat#10569-010) containing 10% fetal bovine serum (FBS) (Gibco, USA, Cat#10099-141) was added to terminate the digestion process. The suspension was filtered through a 100 µm cell strainer and centrifuged at 2,500 r/min for 15 min at room temperature, then we removed the supernatant, resuspended precipitation by PBS (Gibco, USA, Cat# SH30256.01), and filtered by 70 and 40 µm filters in sequence, next centrifuged at 2,200 rpm for 12 min. After resuspended precipitation by RPMI 1640 (Gibco, USA, Cat#A10491-01), centrifuged at 2,000 rpm for 10 min, then removed the supernatant, resuspended precipitation by complete culture medium supplemented with 20% FBS, 0.5% CEE (GEMINI, USA, Cat#100-163P), 1% GlutaMax (Gibco, USA, Cat#35050-061), 1% NEAA (Gibco, USA, Cat#11140-050), 1% AA, RPMI 1640, 2.5 µg/µl bFGF (Gibco, USA, Cat#13256-029), next transferred to the culture dish. To separate and purify satellite cells, after 2.5 h, the suspension was transferred to new culture dish which was coated with Matrigel (Corning, BD, USA, Cat#356234) to get rid of the fibroblasts. For differentiation of porcine skeletal muscle satellite cells into myotubes, cells were transferred to differential medium supplemented with 5% horse serum (Gibco, USA, Cat#26050-070), 1% AA, DMEM at 37°C in 5% CO2 when the confluence of the cells reaches 80–90%.

### Quantitative Reverse Transcription Polymerase Chain Reaction Analysis

Heart, liver, spleen, lung, kidney, brain, skeletal muscle, and subcutaneous fat were taken from three different healthy large white male pigs less than 1-week-old. Tissue samples were frozen in liquid nitrogen and stored at −80°C. Total RNA was collected from large white male pigs tissues and porcine skeletal muscle satellite cells by using TRIzol reagent according to the protocol. Next, the NanoDrop 2000 was used to assess the concentration and integrity. Then, reverse transcribed using PrimeScript™ RT reagent kit with gDNA Eraser. Quantitative real-time PCR (QRT-PCR) was performed with SYBR Green PCR Master Mix on Bio-Rad CFX384 Real-Time System (Bio-Rad Laboratories). QRT-PCR analyses were performed at the following conditions: 95°C for 2 min followed by 40 cycles of 95°C 15 s and melting temperature for 15 s. The specific primers were designed with Primer5 software, and the sequences are listed in **Table S1**. The lncRNA MSTRG.59589 and mRNAs were normalized to 18S, and the 2^−ΔΔCt^ method was applied to calculate the relative expression level.

### Rapid Amplification of Complementary Deoxyribonucleic Acid End Amplification, Sequence Detection, and Coding Ability Prediction

To determine the full-length MSTRG.59589 transcripts, 5' and 3' RACE was performed using Takara SMARTer RACE cDNA amplification (Clontech, 634858/59). We isolated porcine skeletal muscle satellite cells from muscle of neonatal pig hind limb. The muscle of neonatal pig hind limb comes from the same neonatal pig. After induction of differentiation for 24 h, the total RNA was extracted from porcine skeletal muscle satellite cells. According to the manufacturer's instructions, we generated RACE-ready cDNA and designed the gene-specific primers for PCR. The sequences were as follows:

5'RACE GSP TCACTGGTAATGAGGGAAATCCTGGGC
5'RACE NGSP CCTCAGTGCCACCCTCCTCCCAGTT​ACTT
3'RACE GSP GCTCACCAGCAGCACATCCAGACCC
3'RACE NGSP GTCCACGAGGAGCACCTGGCACC

First, the initial PCR product was amplified from RACE-ready cDNA using the 5'RACE gene specific primer (GSP) and 3'RACE GSP primer in the touchdown program, respectively. Then, in order to increase the specificity of race products, 5'RACE NGSP and 3'RACE NGSP primers were used for nested PCR using the initial RCR products as templates. The PCR product was separated on 1% agarose gels, and the bands were extracted and inserted into the PMD-19T vector, positive colonies were selected for sequencing. Sequences were aligned with BLAST in the NCBI standard nucleotide BLAST.

After getting the full length of MSTRG.59589, the Coding potential calculator (CPC) (Kong et al., 2007) and Coding Potential Assessment Tool (CPAT) (Wang et al., 2013) was used to predict the coding potential of MSTRG.59589.

### Nuclear and Cytoplasmic Ribonucleic Acid Fractionation

The nuclear and cytoplasmic RNAs were extracted from porcine skeletal muscle satellite cells during proliferative and differentiation phases. In brief, cells were washed twice with precooled PBS, and centrifuged at 500 g for 3 min. The supernatant was discarded and the precipitation was gently resuspended in 0.2 ml lysis buﬀer [50 mM Tris-HCl pH 8.0 (Biosharp, Cat#BL538A), 140 mM NaCl, 1.5 mM Mgcl_2_, 0.5% IGEPALH CA-630 (Sigma-Aldrich, Cat#I8896), 1 U/µl RNase inhibitor, 1 mM DTT (Avantor, Cat#0281-5G)]. The mixture was placed on ice for 5 min, and then centrifuged at 500×g at 4°C for 3 min. The supernatant was collected to a fresh 1.5 ml RNase-free microcentrifuge tube as the cytoplasmic fraction and centrifuged at 14,000 rpm for 4 min. The remaining pellet was washed with 0.2 ml lysis buffer twice and is corresponding to nuclear fraction. All nuclear and cytoplasmic RNA was extracted with 1 ml TRIzol.

### Antisense Oligonucleotide Synthesis, Plasmid Construction, and Cell Transfection

**Figure 1:**
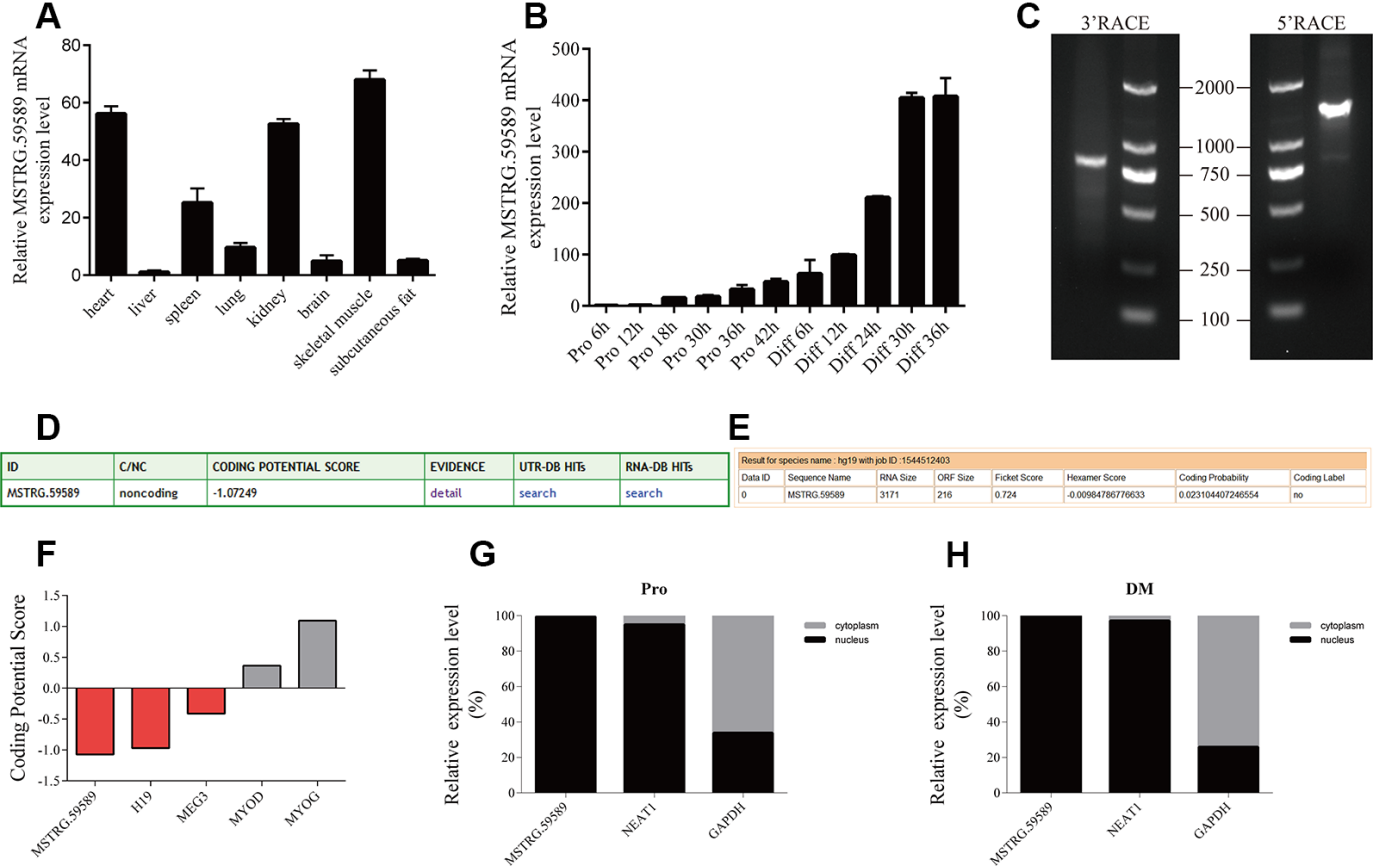
Characterization of long noncoding ribonucleic acid MSTRG.59589. **(A)** Quantitative reverse transcription PCR (QRT-PCR) result showing that MSTRG.59589 has higher expression in the heart, kidney, and skeletal muscle than in other tissues. Mean values ± s.e.m., *n* = 3, *P < 0.05. **(B)** QRT-PCR result showing that the expression level of MSTRG.59589 increases continuously from satellite cell proliferation to differentiation. **(C)** 5′ and 3′ RACE assays were performed to determine the full-length cDNA of MSTRG.59589. **(D)** Coding potential calculator (CPC) was used to predict the coding potential of MSTRG.59589. **(E)** Coding Potential Assessment Tool (CPAT) was used to predict the coding potential of MSTRG.59589. **(F)** Prediction of coding potential using CPC; H19 and MEG3 served as noncoding RNA controls, while MYOD and MYOG served as coding RNA controls. **(G, H)** Distribution of MSTRG.59589 in the nucleus and cytoplasm during proliferation **(G)** and differentiation **(H)**; here, NEAT1 and GAPDH were used as nuclear and cytoplasmic controls, respectively.

Because lncRNA MSTRG.59589 is mainly expressed in the nucleus (**Figures 1G, H**), we designed ASO (antisense oligonucleotide) specifically to knockdown of MSTRG.59589. The website for designing the ASO is http://sfold.wadsworth.org/cgi-bin/index.pl and the ASO synthesized by Sangon Biotech (Wuhan, China). The small interfering RNAs (siRNAs) for knockdown of *PALLD* were designed and synthesized by GenePharma (Suzhou, China). The ASO and siRNA oligo sequences are shown in **Table S2**. Recombinant construct for the overexpression of MSTRG.59589 (PZW1-MSTRG.59589) was obtained by cloning MSTRG.59589 cDNA into the EcoR1 and Kpn1 sites of PZW1-snoVector (Shanghai Academy of Life Sciences, China) (Yin et al., 2015). The primer sequences were listed in **Table S2**. To determine the effects of MSTRG.59589 on differentiation of satellite cells, satellite cells were transfected with MSTRG.59589 ASO, NC (negative control), MSTRG.59589 overexpression vector (PZW1-MSTRG.59589), or control vector (PZW1) using Lipofectamine 2000 (Invitrogen, Life Technologies, USA, Cat#11668019) until the cell density reached 80∼90% in the six-well plates. The final concentrations used for ASO and plasmid were 100 nM and 1.0 µg/ml, respectively.

### Western Blot Assays

Satellite cells were harvested and lysed in RIPA buffer with 1% PMSF on ice. The total protein concentration of the extract was tested by PerkinElmer VICTOR x2 multilabel plate reader using Enhanced BCA Protein Assay Kit (Beyotime, P0009). After that, the supernatant was heated at 95°C for 5 min in 5× sodium dodecyl sulfate (SDS) sample loading buffer. Equal amounts of cells lysate were resolved by 10% SDS-PAGE and transferred onto polyvinylidene fluoride (PVDF) membrane and they were incubated with primary antibody at 4°C overnight. The primary antibody includes anti-(myosin heavy chain antibody) MYHC (Millipore, China, 1:3,000 dilution), anti-tubulin (Millipore, China, 1:2,000 dilution). Then, the PVDF membrane incubated with horseradish peroxidase conjugated secondary antibody (1:4,000) for 1 h at 37°C. Enhanced chemiluminescence substrates was used to visualize signals (Beyotime, China, Cat#P0018A). The fold changes in protein levels were normalized to β-tubulin for quantitative analysis by ImageJ software (https://imagej.nih.gov/ij/).

### Immunofluorescence Staining

After transfection and differentiation of skeletal muscle satellite cells, the cells were washed with PBS two times and fixed in 4% paraformaldehyde for 15 min, then washed with PBS two times and incubated in ice-cold 0.3% Triton X-100 for 10 min, followed by two washes with PBS. Next, the cells were incubated with blocking solution (3%BSA, 0.3% TritonX-100, 10%FBS complemented with PBS) for 2 h and incubated with 1:1,000 diluted MYHC antibodies overnight at 4°C. Then washed cells with PBS two times, the cells were incubated with Alexa 594-labeled anti-mouse antibodies (Antgene, China, ANT029) at room temperature for 1 h. Lastly, the cells were stained with Hoechst 33342 (Sanofi-Aventis, Germany, C1022) for 10 min and washed with PBS twice. The cell nuclei were stained with 4′,6-diamidino-2-phenylindole in the dark. All images were acquired by Leica SP8 confocal microscope. The fold changes for quantitative analysis by ImageJ software ( https://imagej.nih.gov/ij/).

### Ribonucleic Acid Extraction and Transcriptome Sequencing

After knockdown of MSTRG.59589, a total of 12 RNA samples were isolated from porcine skeletal muscle satellite cells at the differentiation 30 and 40 h time points by TRIzol reagent according to the manufacturer's instructions. NanoDrop, Qubit 2.0, and Agilent 2100 methods were used to detect the purity, concentration, and integrity of RNA samples to ensure the use of qualified samples for transcriptome sequencing. After the library was constructed, the concentration and insert size of the library were determined using Qubit 2.0 and Agilent 2100, respectively. The QRT-PCR method was used to accurately quantify the effective concentration of the library. Finally, the transcriptome sequencing was implemented on the Illumina HiSeq X-ten platform (Biomarker, China), and PE150 reads were generated. The raw sequence data files discussed in this experiment have been deposited in Sequence Read Archive (SRA) and SRA ID is PRJNA575967 (https://dataview.ncbi.nlm.nih.gov/object/PRJNA575967), (accession numbers is 30 h ASO at SRR10257656, SRR10257650, SRR10257647; 30 h NC at SRR10257646, SRR10257645, SRR10257655; 40 h ASO at SRR10257654, SRR10257653, SRR10257652; 40 h NC at SRR10257651, SRR10257649, SRR10257648).

### Differential Expression Analysis

The quality of obtained sequence data was evaluated using FastQC software (Xiao et al., 2015) and trimmed using the Trimmomatic tool (version0.3.2). Then, the clean reads were mapped to the pig reference genome (Sus Scrofa11.1) utilizing HISAT2 (version2.0.1) (Pilcher et al., 2015). StringTie (version 1.3.4) was used to assemble RNA-Seq alignments into potential transcripts (Pertea et al., 2016). HTSeq-count (version 0.9.1) was used to count reads mapped to the genome and the annotation file (Anders et al., 2015). Subsequently, differentially expressed genes were identified using the R packages DEGseq2 (Wang et al., 2010). Genes with FDR less than 0.05 and |log2 (fold change) |(|log2FC|) greater than 0 were assigned as differentially expressed. Three comparison libraries were performed using the data obtained from RNA-Seq, including NC groups: 30 h knockdown—NC, 40 h—NC, 30 h NC—40 h NC. Finally, the differentially expressed genes were used to perform Gene Ontology (GO) enrichment analysis and Kyoto Encyclopedia of Genes and Genomes (KEGG) pathway analysis by using DAVID (https://david.ncifcrf.gov/) and integrated differential expression and pathway analysis (iDEP) (http://bioinformatics.sdstate.edu/idep/). Differentially expressed genes were considered to be significantly enriched for GO and KEGG terms with p < 0.05.

## Results

### Molecular Characteristics of the Long Noncoding Ribonucleic Acid MSTRG.59589

To identify the potential role of MSTRG.59589 in porcine tissues, we examined the expression pattern of MSTRG.59589 in different tissues (heart, liver, spleen, lung, kidney, brain, skeletal muscle, and subcutaneous fat) of large white male pigs less than 1-week-old *via* QRT-PCR. We found that MSTRG.59589 has significantly higher expression levels in the heart, kidney, and skeletal muscle than in other tissues (**Figure 1A**). As the proliferation and differentiation of porcine skeletal muscle satellite cells continued, the expression level of MSTRG.59589 increased and reached peak expression 36 h after the beginning of differentiation. This result indicates that MSTRG.59589 is likely to play a role in the late differentiation stage (**Figure 1B**).

We obtained the full-length sequence of MSTRG.59589, which is 3,171 bp long (GenBank MN607221), by 5′ and 3′ rapid amplification of cDNA ends (RACE) (**Figure 1C**). The CPC (**Figure 1D**) and CPAT (**Figure 1E**) prediction indicated that MSTRG.59589 has no coding potential. The coding potentials of the coding genes MYOD and MYOG, as well as those of noncoding genes H19 and MEG3, were predicted using CPC. The prediction results were consistent with existing reports, indicating good accuracy (**Figure 1F**). The subcellular localization of lncRNA determines its action position and mechanism (Chen, 2016). We performed nuclear and cytoplasmic separation experiments in satellite cells during the proliferation and differentiation phases. QRT-PCR results indicated that the MSTRG.59589 transcript is located in the nucleus during proliferation and differentiation (**Figures 1G, H**).

### MSTRG.59589 Promotes the Differentiation of Porcine Skeletal Muscle Satellite Cells

**Figure 2:**
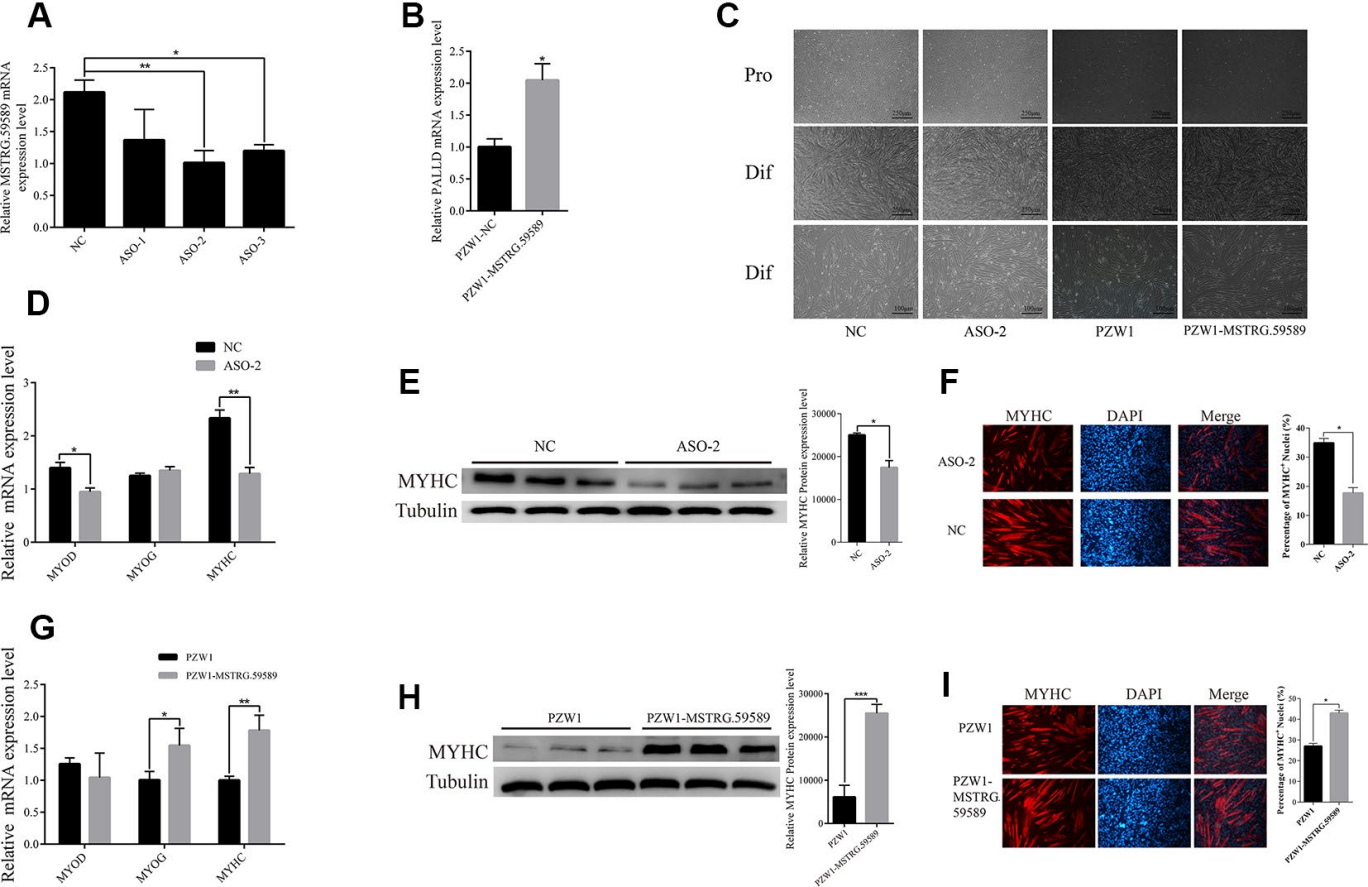
MSTRG.59589 promotes the differentiation of porcine skeletal muscle satellite cells. **(A)** Quantitative reverse transcription PCR (QRT-PCR) results showing that MSTRG.59589 steady-state levels are significantly (52%) decreased in cell treated with antisense oligonucleotide-2. **(B)** QRT-PCR results showing that MSTRG.59589 is successfully significantly overexpressed by PZW1-MSTRG.59589. **(C)** Knockdown and overexpression of MSTRG.59589 inhibited and promoted satellite cell differentiation, respectively. Pro, proliferation; Dif, differentiation. **(D)** QRT-PCR results showing that MSTRG.59589 knockdown significantly decreases the mRNA expression levels of MYOD and MYHC but does not affect MYOG mRNA expression. **(E)** Western blot analysis results showing that MSTRG.59589 knockdown significantly decreases the protein expression levels of MYHC. **(F)** MYHC immunofluorescence staining showing that knockdown of MSTRG.59589 inhibits satellite cell differentiation. QRT-PCR **(G)**, Western blot **(H)**, and immunofluorescence staining **(I)** results showing that overexpression of MSTRG.59589 promotes satellite cell differentiation.

To reveal the role of lncRNA MSTRG.59589 in skeletal muscle cell development, we performed knockdown and overexpression experiments in porcine skeletal muscle satellite cells. Considering the subcellular distribution of MSTRG.59589, we designed and synthesized three independent phosphonothioate antisense oligonucleotides (ASO) for MSTRG.59589 knockdown and constructed the recombinant plasmid PZW1-MSTRG.59589 for MSTRG.59589 overexpression. QRT-PCR results showed that MSTRG.59589 is significantly inhibited by ASO-2 and successfully overexpressed in the PZW1-MSTRG.59589 group in differentiation medium (DM) (**Figures 2A, B**). When MSTRG.59589 was knocked down, the myotubes in DM exhibited smaller sizes than those in the control group, and the progress of satellite cell differentiation was suppressed compared with that in the control group (**Figure 2C**). As expected, MSTRG.59589 overexpression revealed results contrasting those of knockdown (**Figure 2C**). Hence, we speculate that MSTRG.59589 may be related to the differentiation of porcine skeletal muscle satellite cells. QRT-PCR, Western blot, and immunofluorescence staining were performed to detect the differentiation-related marker genes MYOD, MYOG, and MYHC and assess changes in cell differentiation ability. MSTRG.59589 knockdown significantly inhibited the differentiation of satellite cells, as demonstrated by the decreased mRNA and protein expression of the differentiation-related marker genes MYOD and MYHC (**Figures 2D–F**). However, no significant change in the expression of the MYOG gene was observed after MSTRG.59589 knockdown. As expected, MSTRG.59589 overexpression significantly increased the expression levels of MYOG and MYHC at the mRNA and protein levels, but no significant changes were observed in the expression of the MYOD gene (**Figures 2G–I**). Thus, our experiments suggest that MSTRG.59589 may promote satellite cell differentiation.

### Detection of Changes in Differentially Expressed Genes Before and After Knockdown of MSTRG.59589 by Ribonucleic Acid Sequencing Data Analysis

**Figure 3:**
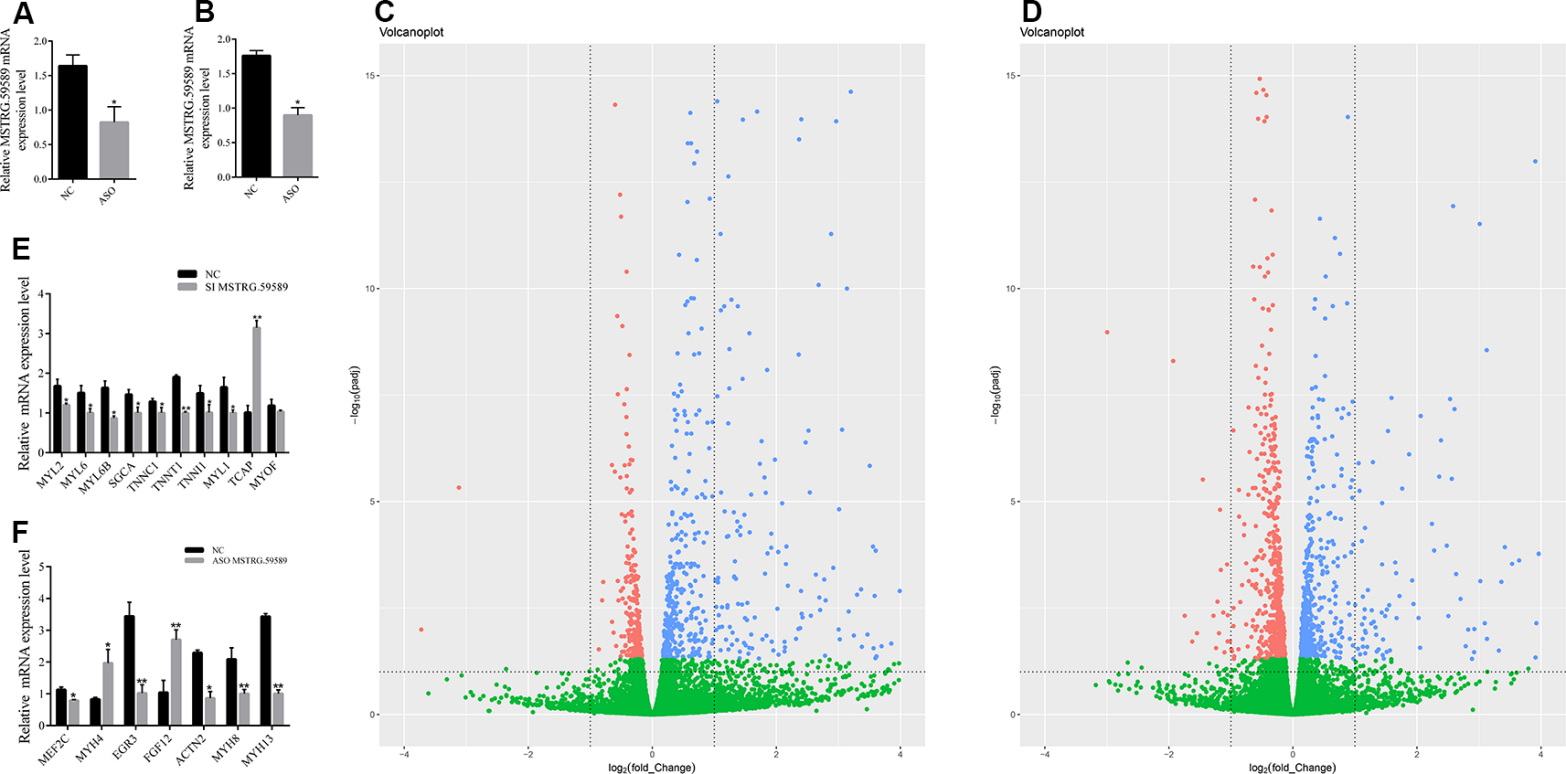
Analysis of differentially expressed genes and quantitative reverse transcription PCR (QRT-PCR) validation. Interference efficiency of antisense oligonucleotide (ASO) 30 **(A)** and 40 h **(B)** after induction of cell differentiation. Volcano figure of differentially expressed genes between the MSTRG.59589 knockdown (ASO-MSTRG.59589) and control (NC) groups 30 h **(C)** and 40 h **(D)** after induction of cell differentiation, red: down-regulated genes, blue: up-regulated genes, green: no differentially. QRT-PCR was performed to validate the differentially expressed genes determined from RNA sequencing data 30 h **(E)** and 40 h **(F)** after induction of cell differentiation.

To identify the potential downstream target genes of MSTRG.59589 more accurately, we transfected ASO to knock down MSTRG.59589 in satellite cells. The cells were then collected 30 and 40 h after inducing cell differentiation. QRT-PCR results indicated that MSTRG.59589 expression is significantly reduced (**Figures 3A, B**). Illumina HiSeq PE150 sequencing technology was used to perform RNA-seq of the 12 cDNA libraries of the above cells and control groups; in total, we obtained over 35 million raw reads from each library (**Table S3**). After removing low-quality sequences, clean reads comprised more than 98% of the raw data (**Table S3**). Next, we aligned all clean reads to the porcine Sscrofa11.1 reference genome and found that more than 96% clean reads could be uniquely mapped to the genome (**Table S3**). We analyzed the expression levels of all protein coding genes (**Table S4**), cufflinks were used to transcript assembly, and DEseq software was used to identify differentially expressed genes. Compared with those in the control group (30 h NC), 480 genes were up-regulated and 231 genes were down-regulated 30 h after induction of satellite cell differentiation (**Figure 3C**, **Table S5**). A total of 1,126 genes were found to be differentially expressed 40 h after MSTRG.59589 knockdown, including 527 up-regulated and 599 down-regulated genes (**Figure 3D**, **Table S6**). Among the differentially expressed genes screened during satellite cell differentiation at 30 and 40 h, we randomly selected nine (30 h) (up-regulated gene: TCAP, down-regulated genes: MYL2, MYL6, MYL6B, SGCA, TNNC1, TNNT1, MYL1) and seven (40 h) (up-regulated genes: MYH4, FGF12, down-regulated genes: MEF2C, EGR3, ACTN2, MYH8, MYH13) muscle-development-related genes for QRT-PCR experiments to verify the accuracy of the RNA-seq data and found results consistent with those from RNA-seq (**Figures 3E, F**).

### Differentially Expressed Genes During Differentiation of Porcine Skeletal Muscle Satellite Cells

**Figure 4:**
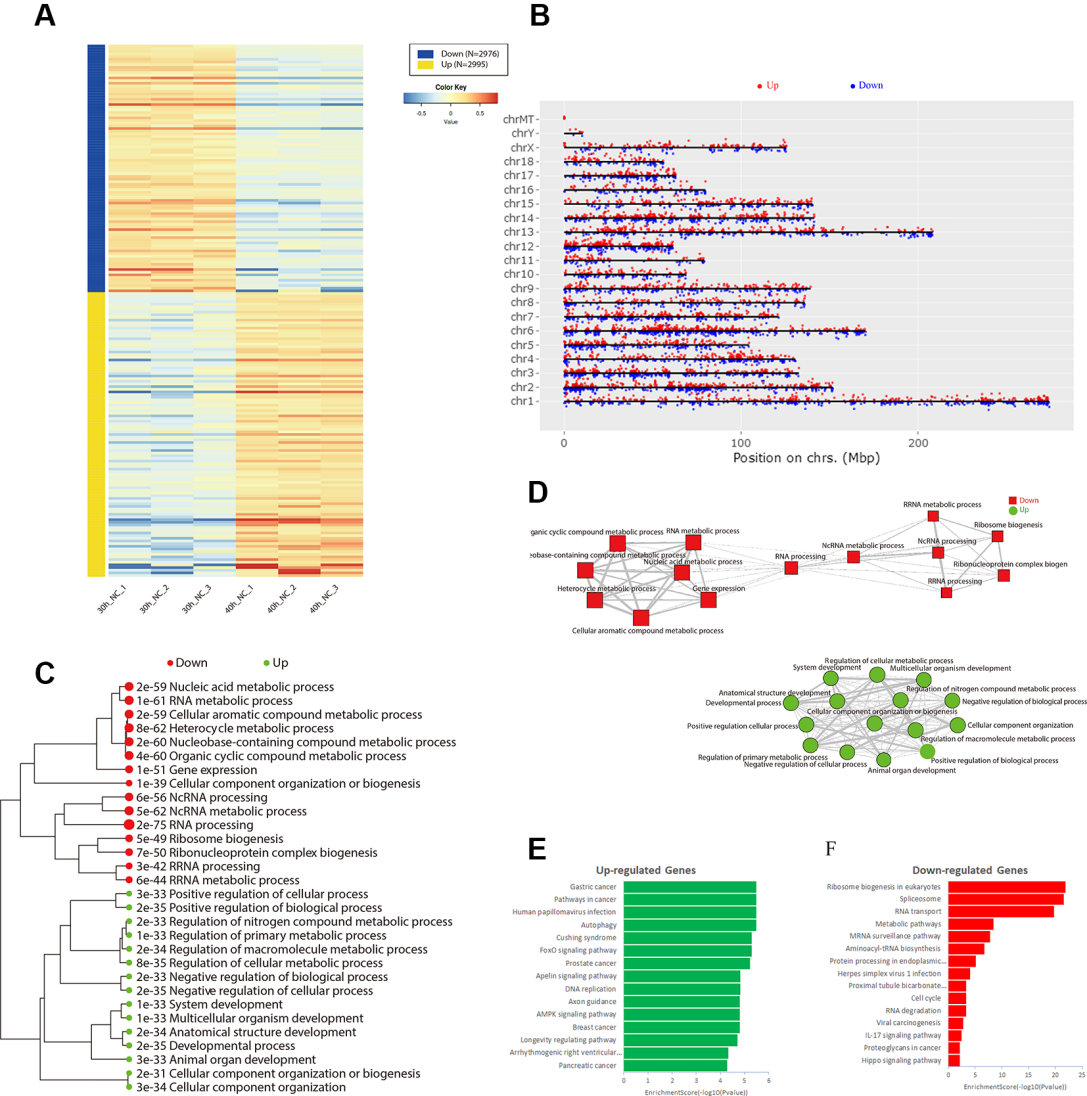
Identification of differentially expressed genes during differentiation of porcine skeletal muscle satellite cells. **(A)** Heat map of differentially expressed genes between the 30 and 40 h negative control (NC) groups. **(B)** Distribution of differentially expressed genes in all chromosomes. Hierarchical clustering tree **(C)** and networks **(D)** for Gene Ontology enrichment of up-regulated (green) and down-regulated (red) genes in 30 and 40 h NC groups. Kyoto Encyclopedia of Genes and Genomes pathway analysis of up-regulated **(E)** and down-regulated **(F)** genes in the 30 and 40 h NC groups.

To identify the genes involved in porcine skeletal muscle satellite cell differentiation, we analyzed differentially expressed genes between 30 and 40 h after induction of porcine skeletal muscle satellite cell differentiation. A total of 5,971 genes were identified (**Table S7**). Among them, 2,995 genes were up-regulated and 2,976 genes were down-regulated (**Figure 4A**). The expression and function of the adjacent genes may be associated with the differentially expressed genes identified above; hence, we analyzed the distribution of differentially expressed genes in all chromosomes (**Figure 4B**). Several differentially expressed gene clusters are distributed in each chromosome region; for example, a 4.6 Mbps region on Chr.12q10 contains five up-regulated genes (KCNJ16, ABCA8, RGS9, CACNG1, and CD79B). We constructed a hierarchical clustering tree (**Figure 4C**) and a network of GO terms (**Figure 4D**) using iDEP (http://bioinformatics.sdstate.edu/idep/). Up-regulated genes were mainly related to the developmental process, positive regulation of biological process, and negative regulation of the cellular process, while down-regulated genes were associated with heterocyclic metabolic processes, ncRNA metabolic processes, and RNA processing (**Table S8**). Kyoto Encyclopedia of Genes and Genomes (KEGG) pathway analysis indicated that up-regulated genes were mainly enriched in breast cancer, the AMPK signaling pathway, and axon guidance (**Figure 4E**), while down-regulated genes were significantly enriched in the Hippo signaling pathway, proteoglycans in cancer, and IL-17 signaling pathway (**Figure 4F**, **Table S9**).

### Gene Ontology and Kyoto Encyclopedia of Genes and Genomes Enrichment of Differentially Expressed Genes in the 30 and 40 H Satellite Cell Differentiation Groups

**Figure 5:**
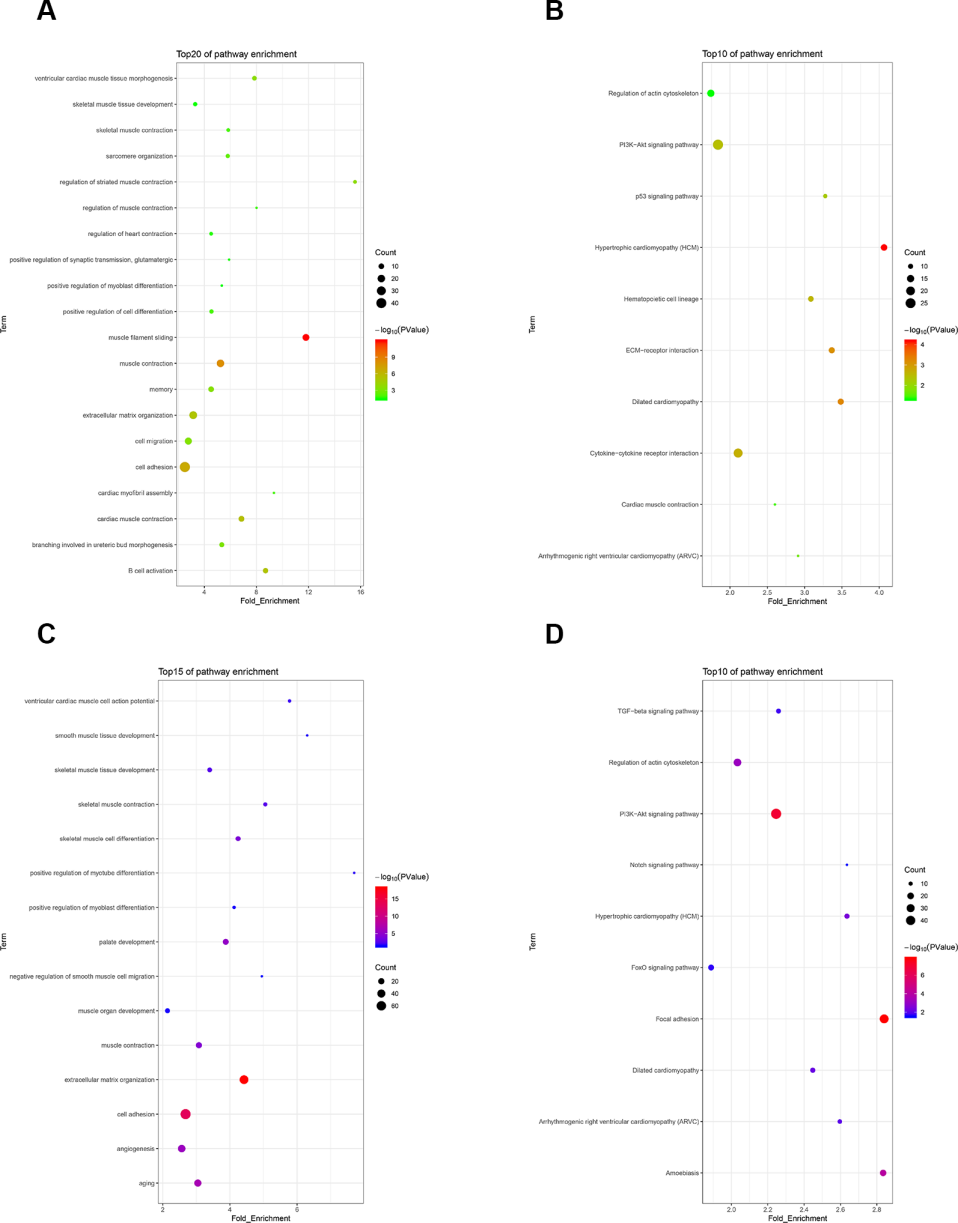
Gene Ontology (GO) and Kyoto Encyclopedia of Genes and Genomes (KEGG) enrichment analyses of differentially expressed genes. GO **(A)** and KEGG **(B)** pathway analyses of differentially expressed genes 30 h after induction of cell differentiation. GO **(C)** and KEGG **(D)** pathway analyses of differentially expressed genes 40 h after induction of cell differentiation.

**Figure 6:**
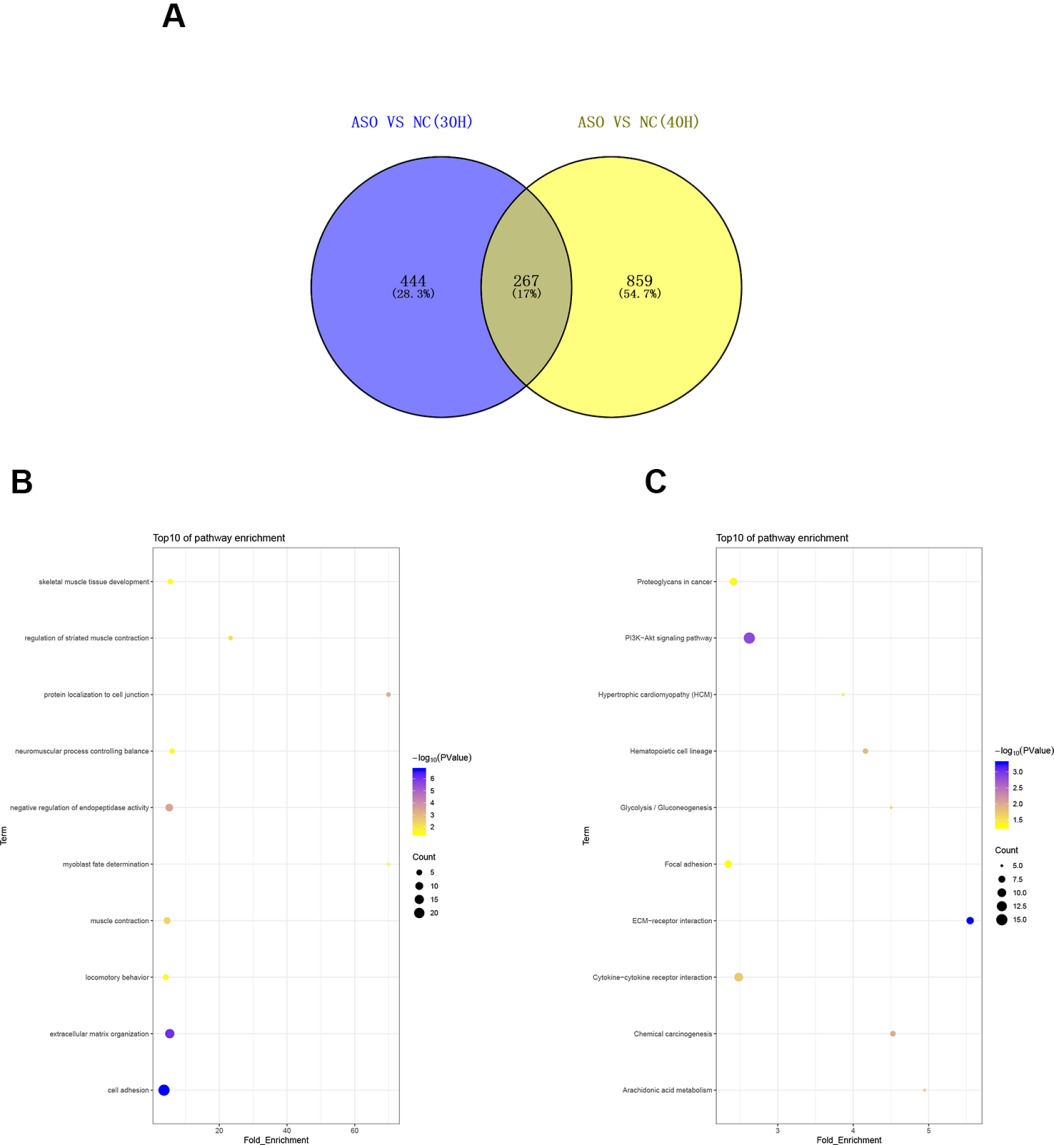
Analysis of common differentially expressed genes between the 30 and 40 h cell differentiation groups. **(A)** Venn diagram analysis of commonly differentially expressed genes at 30 and 40 h after induction of cell differentiation. Gene Ontology **(B)** and Kyoto Encyclopedia of Genes and Genomes **(C)** pathway analyses of commonly differentially expressed genes.

To investigate the functions of differentially expressed genes, we performed GO and KEGG analyses by using DAVID bioinformatics online tools. In the 30 h cell differentiation group, GO analysis showed that 599 of 698 differentially expressed genes significantly participated in 110 biological processes (**Table S10**), and many were found to be involved in muscle-development-related biological processes, such as regulation of striated muscle contraction, muscle filament sliding, and cardiac muscle contraction (**Figure 5A**). KEGG analysis indicated that 282 of 698 differentially expressed genes significantly participated in 20 pathways (**Table S10**). The differentially expressed genes were mainly enriched in hypertrophic cardiomyopathy (HCM), dilated cardiomyopathy, and ECM–receptor interaction (**Figure 5B**). In the 40 h cell differentiation group, GO analysis showed that 969 of 1,098 differentially expressed genes significantly participated in 219 biological processes (**Table S10**), and many were found to be involved in muscle-development-related biological processes, such as positive regulation of myotube differentiation, smooth muscle tissue development, and ventricular cardiac muscle cell action potential (**Figure 5C**). KEGG analysis indicated that 435 of 1,098 differentially expressed genes significantly participated in 37 pathways (**Table S10**). The differentially expressed genes were mainly enriched for focal adhesion, amoebiasis, and HCM (**Figure 5D**). We constructed a Venn diagram to further analyze differentially expressed genes in the 30 and 40 h satellite cell differentiation groups. As shown in **Figure 6A**, 267 differentially expressed genes were found to be common between the two comparison groups. We performed GO and KEGG enrichment analyses of these 267 differentially expressed genes and found that many of them significantly participated in 41 biological processes and 10 pathways (**Table S10**). As expected, muscle-development-related pathways, such as myoblast fate determination and HCM, were among the top 10 function clusters determined *via* GO and KEGG analyses (**Figures 6B, C**). From the above results, we speculate that lncRNA MSTRG.59589 may be involved in porcine skeletal muscle satellite cell differentiation.

### Relationship Between the Long Noncoding Ribonucleic Acid MSTRG.59589 and the Target Gene *PALLD*

**Figure 7:**
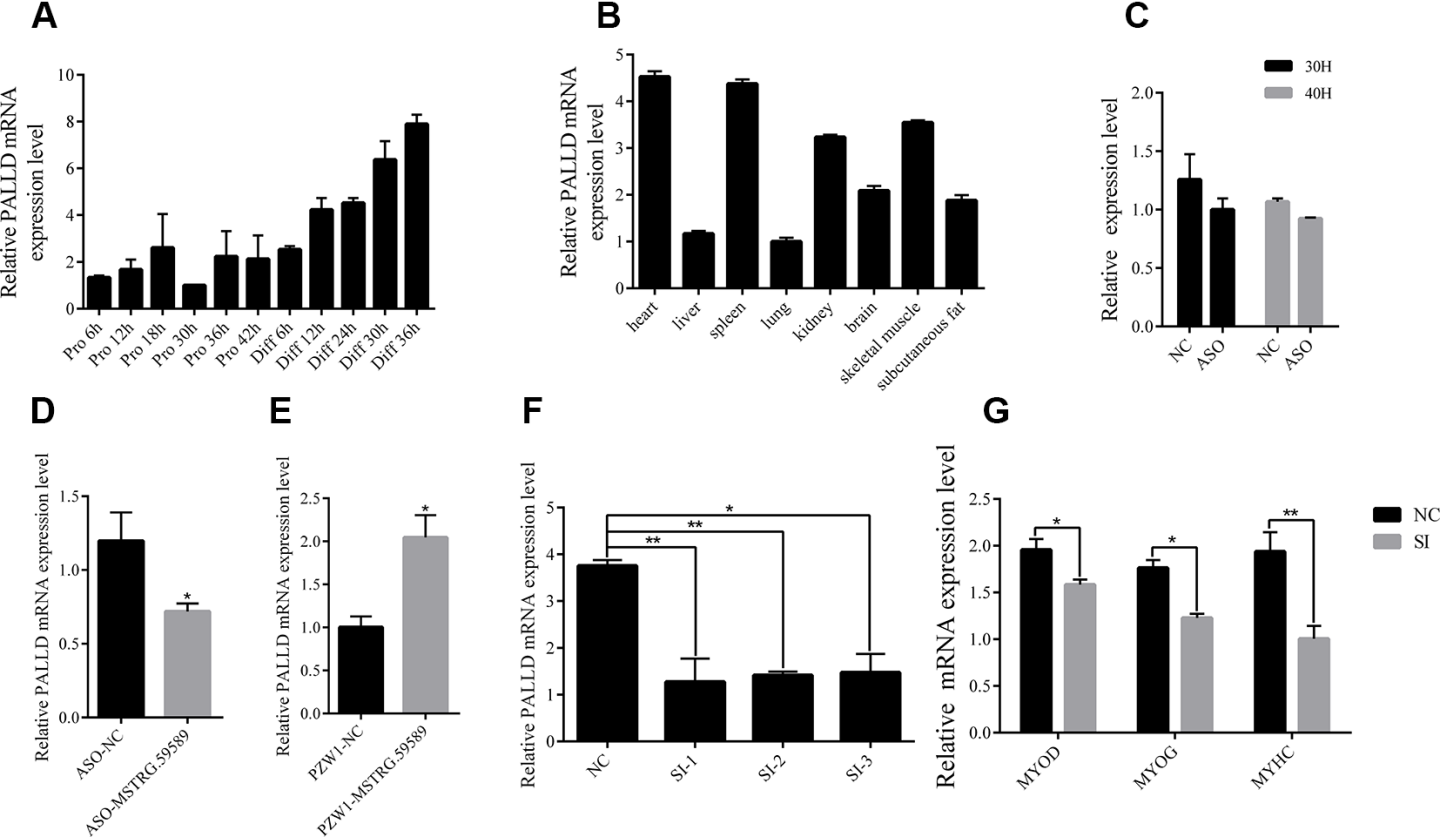
MSTRG.59589 promoted muscle differentiation by enhancing the expression of *PALLD*. **(A)** Quantitative reverse transcription PCR (QRT-PCR) results showing that the expression level of the *PALLD* increases continuously from satellite cell proliferation to differentiation. **(B)** QRT-PCR results showing that *PALLD* is more highly expressed in the heart, skeletal muscle, and spleen than in other tissues. RNA sequencing and QRT-PCR results showing that the expression of *PALLD* is down-regulated **(C, D)** or up-regulated **(E)** after knockdown or overexpression of MSTRG.59589. **(F)** QRT-PCR results showing that *PALLD* is successfully significantly inhibited by SI-2. **(G)** QRT-PCR results showing that *PALLD* knockdown significantly decreases the messenger RNA expression levels of MYOD, MYOG, and MYHC.

According to the previous studies, lncRNAs can influence biological processes by regulating the expression of its neighboring genes, and lncRNA MSTRG.59589 (sus scrofa11.1, Chr 14, 20806080 to 20809250) is an antisense transcription of the second intron of the *PALLD* gene. *PALLD* could affect the number and size of actin bundles, as well as muscle cell proliferation and differentiation (Boukhelifa et al., 2003; Nguyen et al., 2014). Therefore, we hypothesized that MSTRG.59589 may be involved in the differentiation of skeletal muscle satellite cells by regulating *PALLD* expression. The mRNA expression level of *PALLD* was gradually up-regulated as the proliferation and differentiation of skeletal muscle satellite cells progressed (**Figure 7A**). Tissue expression profiles showed that *PALLD* is widely expressed in various tissues, especially in the heart, spleen, and skeletal muscle (**Figure 7B**). To clarify whether *PALLD* is modulated by MSTRG.59589, we detected its mRNA level when MSTRG.59589 is knocked down and overexpressed. After MSTRG.59589 was inhibited, the mRNA expression level of *PALLD* declined remarkably; by contrast, after MSTRG.59589 was overexpressed, it increased remarkably (**Figures 7C–E**). To detect the effects of *PALLD* on the differentiation of skeletal muscle satellite cells, we designed three RNA interference oligonucleotides to knock down the *PALLD* gene. The SI-2 fragments specifically demonstrated the highest interference (**Figure 7F**). We examined the effect of down-regulated *PALLD* on porcine skeletal muscle satellite cell differentiation, and QRT-PCR results showed that the mRNA levels of MYOD, MYOG, and MYHC decrease, thus suggesting that down-regulation of *PALLD* could inhibit porcine skeletal muscle satellite cell differentiation (**Figure 7G**). Consequently, our study demonstrates that alteration of MSTRG.59589 may mediate decreases in *PALLD* and affect porcine skeletal muscle satellite cell differentiation.

## Discussion

**Figure 8:**
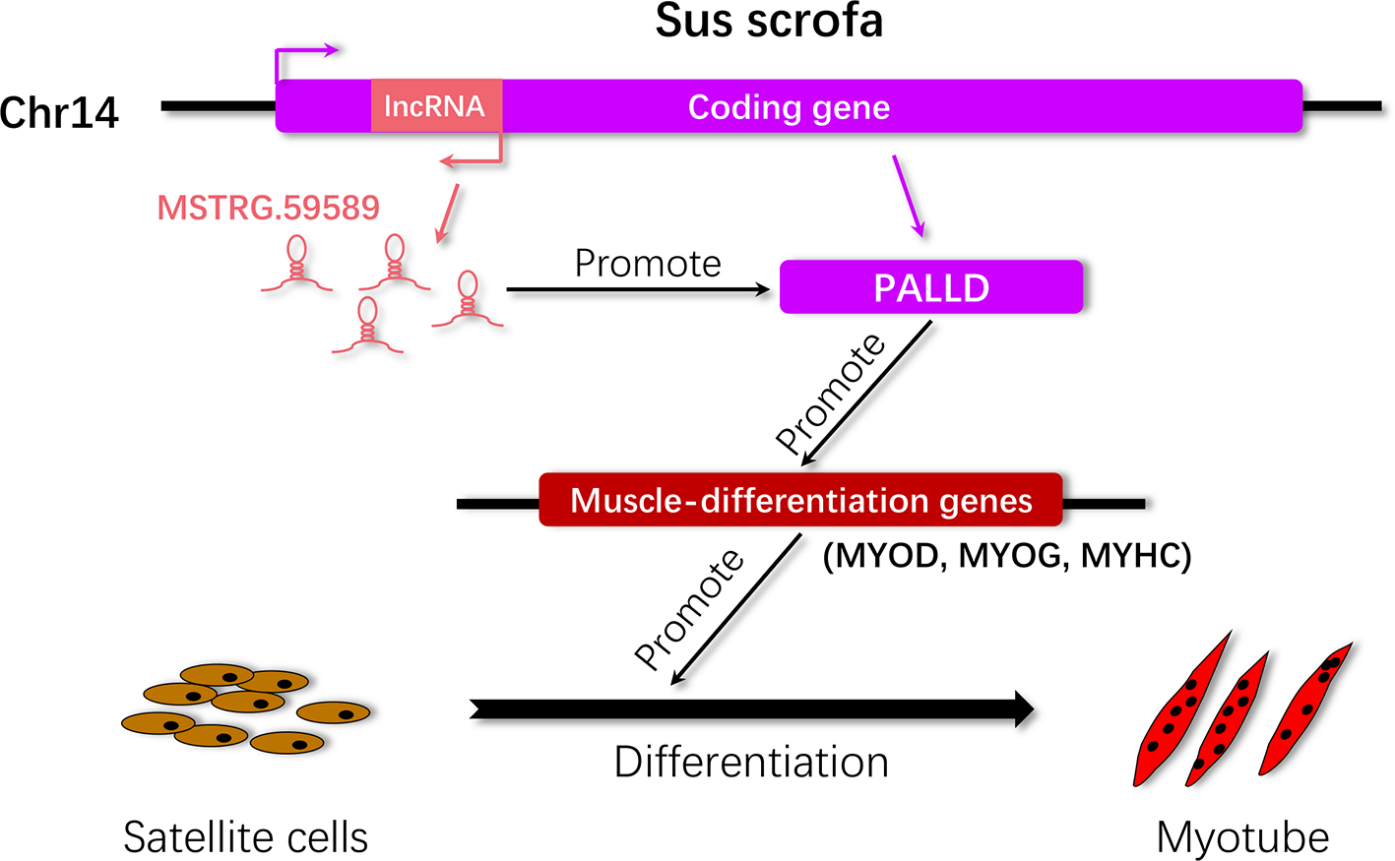
Proposed working model by which MSTRG.59589 regulates satellite cell differentiation. LncRNA MSTRG.59589 is an antisense transcript of *PALLD* that can increase the expression level of the latter, leading to the up-regulated expression of the muscle differentiation genes MYOD, MYOG, and MYHC and ultimately promoting satellite cell differentiation.

Porcine satellite cells are known not only for their essential role in muscle formation and development but also for their excellent self-renewal ability after injury. LncRNAs play an essential role in regulating muscle cell development (Neguembor et al., 2014). In the current study, we first identified the molecular feature and expression patterns of the lncRNA MSTRG.59589 through molecular biology experimental techniques and bioinformatics methods. Then, we performed expression analysis of this lncRNA in porcine tissues and skeletal muscle satellite cells to reveal its expression patterns. We performed transcriptome analysis of MSTRG.59589 knockdown in porcine skeletal muscle satellite cells at two differentiation time points (30 and 40 h) and characterized differentially expressed genes and pathways to verify how the lncRNA affects target genes and pathways and phenotypic changes in porcine skeletal muscle satellite cells. We explored the molecular mechanisms responsible for the target gene and pathway regulation of lncRNA MSTRG.59589 its effects the differentiation of porcine skeletal muscle satellite cells. Our present study preliminarily reveals that MSTRG.59589 may promote the differentiation of skeletal muscle satellite cells by increasing the expression of *PALLD* (**Figure 8**).

In our study, we obtained the full-length sequence of MSTRG.59589 by RACE. MSTRG.59589 has a full length of 3,171 bp and a distinct polyA tail structure. The protein coding ability of MSTRG.59589 was predicted by CPC and CAPT software, and results showed that MSTRG.59589 is a noncoding RNA. Then, we discovered that, among the different tissues (heart, liver, spleen, lung, kidney, brain, skeletal muscle, and subcutaneous fat) of large white male pigs, the expression level of MSTRG.59589 is the highest in the skeletal muscle. After isolation and culture of porcine skeletal muscle satellite cells, during the proliferation and differentiation of cells, we collected cell samples every 6 h and examined the expression changes of MSTRG.59589. Results showed that MSTRG.59589 demonstrates a gradual increase in the expression of satellite cells from proliferation to differentiation. This evidence implies that MSTRG.59589 probably has a potential function in muscle development. In addition, distinct subcellular enrichment of lncRNAs could lead to diverse functions and mechanisms of action (Chen, 2016). We confirmed that MSTRG.59589 is mainly expressed in the nucleus, thus indicating that it may work at the transcriptional level or regulate chromatin remodeling (Chen and Carmichael, 2010).

To explore the effects of MSTRG.59589 on satellite cell differentiation, we performed knockdown and overexpression experiments on MSTRG.59589, which is the only way to study the basic regulating functions of the lncRNA. The results showed that knockdown of MSTRG.59589 could inhibit satellite cell differentiation *in vitro*, whereas MSTRG.59589 overexpression contributes to the acceleration of satellite cell differentiation. To confirm the relevant pathways and downstream genes of MSTRG.59589 affecting skeletal muscle satellite cell differentiation, we constructed an MSTRG.59589 knockdown satellite cell model for transcriptome sequencing. A total of 711 and 1,126 differentially expressed genes were respectively identified 30 and 40 h after transfection of satellite cells with a specific ASO against MSTRG.59589. GO and KEGG enrichment analyses were performed, and a large number of differentially expressed genes were found to be enriched into muscle-development-related pathways. Among the differentially expressed genes at 30 h, the expression levels of MYL2, MYL6, MYL6B, SGCA, TNNC1, TNNT1, TNNI1, and MYL1 were down-regulated, whereas the expression of TCAP was up-regulated. Among the differentially expressed genes at 40 h, MYH4 and FGF12 were up-regulated, whereas MYH8, MYH13, MEF2C, EGR3, and ACTN2 were down-regulated. The MYL1, MYL2, MYL6, and MYL6B genes belong to the myosin light chain family and can regulate the transformation of muscle fiber types, participate in the phosphorylation driving role in the myocardium, and interact with the myosin heavy chain through fine molecular structure rearrangement, thereby regulating the proliferation and differentiation of muscle cells (Zhang et al., 2009; Huang et al., 2015; Sheikh et al., 2015). The TNNC1, TNNT1, and TNNI1 genes are members of the slow-acting troponin family, which are mainly expressed in skeletal muscle, slow muscle, and myocardium and control muscle contraction and relaxation. Some studies have shown that the expression levels of these genes are associated with pig meat quality traits (Pierzchala et al., 2014). The MYH4, MYH8, and MYH13 genes are members of the myosin heavy chain family and are highly conserved and ubiquitous. They can participate in various biological processes, such as cytokinesis, phagocytosis, and muscle contraction (Weiss and Leinwand, 1996). The presence of these differential genes suggests that knockdown of MSTRG.59589 greatly alters the expression of muscle-development-related genes, thus implying its effect on muscle development. Interestingly, during GO and KEGG enrichment analyses, we found that some differential genes are enriched in the actin cytoskeletal pathway. Coincidentally, MSTRG.59589 is transcribed by the second intron of *PALLD*. However, the PALLD gene encodes a cytoskeletal protein that is required for organizing the actin cytoskeleton; hence, we speculate that MSTRG.59589 may regulate muscle differentiation through *PALLD*.

The differentiation of muscle progenitor cells into mature myotubes is a highly ordered process. Initially, mononuclear myoblasts proliferate, irreversibly withdraw from the cell cycle, migrate, and align with each other. Then, the myoblasts fuse to form multinucleated myotubes and terminally differentiate muscle fibers. Skeletal muscle differentiation is required to enhance the actin cytoskeletal rearrangement for complex changes, including maintenance of cell shape, coordination of cell migration, adhesion, fusion, intracellular and extracellular matrix interactions, and muscle contraction, in this biological process, *PALLD* as a molecular scaffold and actin cross-linker, being a component of the actin-containing microfilaments, it is involved skeletal muscle differentiation (Geeves and Holmes, 1999; Parast and Otey, 2000; Goicoechea et al., 2008; Goicoechea et al., 2009). Some research has shown that one of the *PALLD* isoforms may play a scaffolding role in sarcomeric organization (Wang and Moser, 2008). Inactivation of *PALLD* leads to embryonic lethality, and, in an *in vitro* study, the absence of *PALLD* severely interfered with the formation of actin stress fibers, adhesion, and migration of mouse embryonic fibroblasts (Luo et al., 2005). At present, few studies have investigated the effect of *PALLD* on muscle development. Overexpression of *PALLD* in astrocytes reveals an increase in the number and size of actin bundles (Boukhelifa et al., 2003). During myofibroblast differentiation, TGF-β1 induces up-regulation of *PALLD* expression (Ronty et al., 2006). *PALLD* also plays a key role in smooth muscle cell differentiation (Jin et al., 2010; Jin, 2011). After *PALLD* knockdown by siRNA, the migration rate of C2C12 cells slowed down but their proliferation and differentiation abilities were enhanced (Nguyen et al., 2014).

No relevant studies on *PALLD* in porcine skeletal muscle development have been conducted, but *PALLD* is probably involved in the development of porcine skeletal muscle. To explore whether *PALLD* regulates skeletal muscle development as expected, we first examined the expression of *PALLD* gene in various tissues and found that it is a widely expressed gene with high expression levels in the heart, spleen, kidney, and skeletal muscle. Then, we examined its expression changes in various stages of satellite cell proliferation and differentiation and found that its expression level is generally higher in the differentiation stage than in the proliferation stage, thus demonstrating a gradually increasing trend in the former. After knockdown of *PALLD* by siRNA, we detected changes in differentiated marker genes. We found that the expression levels of the differentiated marker genes MYOD, MYOG, and MYHC are significantly down-regulated, which indicates that knockdown of *PALLD* inhibited the differentiation of satellite cells. Therefore, *PALLD* gene exhibits a promoting effect on muscle differentiation. Muscle is formed by the fusion of numerous muscle fibers. The most basic unit of muscle fibers is the sarcomere, which is composed of thin muscle filaments (actin) and thick muscle filaments (myosin). These parallel thick and thin filaments slide across one another to regulate muscle contraction (Lin et al., 2017). *PALLD* can control the number and size of the actin bundles. MYHC is one of the components of myosin, and after knockdown of *PALLD*, its expression level decreases, thus indicating that *PALLD* plays a decisive role in actin and myosin interacting relationship. Therefore, studying the mechanisms among lncRNA MSTRG.59589, *PALLD*, and MYHC can not only elucidate the regulation of lncRNA in muscle differentiation but also provide a theoretical basis for the treatment of muscle weakness and muscle atrophy.

In summary, the identified lncRNA MSTRG.59589 is involved in the differentiation of porcine skeletal muscle satellite cells. Our study reveals that MSTRG.59589 could induce increases in *PALLD* expression level and promote porcine skeletal muscle satellite cell differentiation (**Figure 8**). However, several gaps in our study deserve further exploration. In particular, we are interested in deciphering the molecular mechanisms of MSTRG.59589 affecting *PALLD* expression and porcine skeletal muscle satellite cell differentiation, as well as its functions in animals.

## Data Availability Statement

The raw sequence data files discussed in this experiment have been deposited in SRA and SRA ID is PRJNA575967 ( https://dataview.ncbi.nlm.nih.gov/object/PRJNA575967), (accession numbers is 30h ASO at SRR10257656, SRR10257650, SRR10257647; 30h NC at SRR10257646, SRR10257645, SRR10257655; 40h ASO at SRR10257654, SRR10257653, SRR10257652; 40h NC at SRR10257651, SRR10257649, SRR10257648).

## Ethics Statement

All experimental animal procedures in this study were carried out in accordance with the pre-approved guidelines from Regulation Proclamation No. 5 of the Standing Committee of Hubei People's Congress. The experimental protocols in our study were approved by the Ethics Committee of Huazhong Agricultural University, Wuhan City, Hubei Province, P. R. China.

## Author Contributions

CL conceived and designed the experiments and explained the data. LL analyzed main content of the data with the help of LC, performed the experiment with the help of JL, XC, WL. LL wrote the paper with the help of CL. All authors have reviewed and approved the manuscript.

## Funding 

The work was supported by National Natural Science Foundation of China (NSFC, 31872322) and the Fundamental Research Funds for the Central Universities (2662017PY030).

## Conflict of Interest

The authors declare that the research was conducted in the absence of any commercial or financial relationships that could be construed as a potential conflict of interest.
